# Synchronous colonic adenoma and intestinal marginal zone B-cell lymphoma associated with Crohn’s disease: a case report and literature review

**DOI:** 10.1186/s12885-019-6224-x

**Published:** 2019-10-17

**Authors:** Amal Bennani, Ghizlane Kharrasse, Miry Achraf, Khanoussi Wafa, Ismaili Zahi, Kamaoui Imane, Bouziane Mohamed

**Affiliations:** 1 Department of pathology, Mohamed I University, 30050 Oujda, Morocco; 2 Laboratory of Epidemiology, Clinical Research and Public, Medical School of Oujda, Oujda, Morocco; 3 Department of Gastroenterology, Mohamed I University, 30050 Oujda, Morocco; 4 Department of radiology, Mohamed I University, 30050 Oujda, Morocco; 5 Department of Surgical Oncology, Mohamed I University, 30050 Oujda, Morocco

**Keywords:** Lymphomatous polyposis, MALT, Adenoma, Crohn’s disease

## Abstract

**Background:**

Lymphoma and dysplasia are rare complications of long-standing Crohn’s disease. We report an exceptional case of a synchronous intestinal marginal zone B-cell lymphoma (MALT lymphoma) and colonic adenoma in a Crohn’s disease patient.

**Case presentation:**

A 50-year-old male patient presented with right lower quadrant for the last 9 months. He also had associated weight loss and diarrhea alternating with constipation. Ileo-colonoscopy revealed a pseudopolypoid appearance of the colonic and ileal mucosa with many discontinuous ulcerations with a 3 cm sessile polypoid mass at 17 cm from the anal verge. Histological examination of the polypoid lesion revealed an adenoma with high grade dysplasia, while the biopsies of colonic mucosa showed histologic features of Crohn’s disease. Abdominal computed tomography scan (CT scan) and magnetic resonance imaging (MRI) showed circumferential wall thickening of the colon and ileum, enlarged mesenteric lymph nodes and a sessile polypoid mass of the rectosigmoid junction. The patient was scheduled for an ileocoletectomy with resection of the upper rectum and ileorectostomy.

The histological examination of the resected segment showed histologic features of Crohn’s disease, a recto-sigmoid polyp with high grade.

dysplasia and extensive small lymphocytic infiltrate in both colonic and ileal wall which is strongly stained by CD20 and BCL2. The diagnosis of MALT lymphoma with adenoma on a background of Crohn’s disease was made.

The patient successfully completed 8 cycles of Rituximab+ chlorambucil chemotherapy.

Nowadays the patient is asymptomatic without evidence of lymphoproliferative recurrence 10 months after surgery.

**Conclusion:**

We report the first case in the literature of Malt lymphoma with colonic adenoma associated with Crohn’s disease, and discuss his unique macroscopic and histological features in a patient.

Without immunosuppressive therapy.

## Background

Crohn’s disease is a chronic and idiopathic inflammatory.

disease that has the potential to affect any segment of the gastrointestinal tract from mouth to anus. It is characterized by patchy involvement with transmural inflammation. Although many studies have suggested that patients with Crohn’s disease may have an increased risk of developing lymphoma [1], the relationship between these entities remain unclear [2]. It is also know that patients with Crohn’s disease have increased risk of colorectal carcinoma and dysplasia [3, 4]. The association of colonic lymphoma and adenocarcinoma has rarely been reported in the literature [5], while the coexistence of precursor lesion (adenoma) and lymphoma has never been described in the same specimen. We report the first case in the literature of a synchronous MALT lymphoma and colonic adenoma with high grade dysplasia in a patient with Crohn’s disease.

## Case presentation

In this report, we describe a 50-year-old male patient presented with right lower quadrant for the last 9 months. He also has had associated weight loss (9 kg) and diarrhea alternating with constipation. He was a former smoker (22 pack-years), and current drinker (1–3 drinks per week). He had undergone appendicectomy for appendicitis 25 years earlier with no evidence Crohn’s disease on histology. He also had no familial history of inflammatory bowel disease or cancer.

On examination, he was dehydrated and had no fever. His pulse rate was 90 beats per minute, blood pressure 110/60 mmHg and respiratory rate 16 cycles per minute.

The abdominal examination showed deep tenderness in lower right quadrant, without palpable mass or draining fistula.

### Investigations

Laboratory tests at presentation showed an elevated C-reactive protein level of 32 mg/L and low albumin of 28 g/L. A complete blood count revealed total leukocytes 4800/mm3 and hemoglobin 10.2 g/dl. Other bloodwork was unremarkable.

**Fig. 1:**
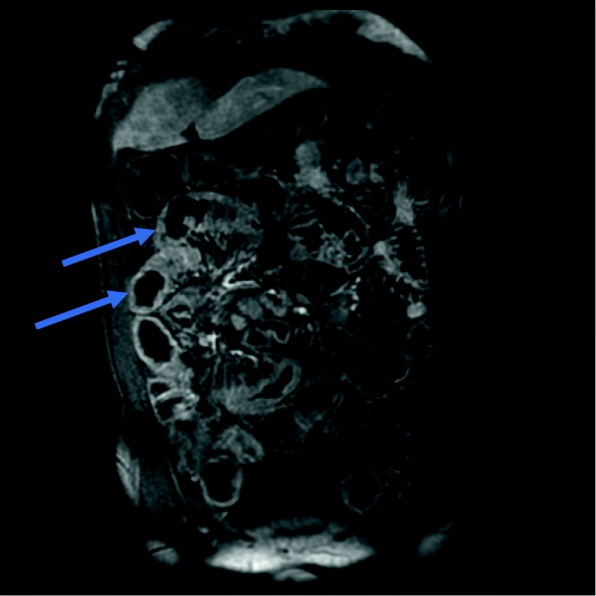
*T1 coronal* section after gadolinium *injection* showing a circumferential wall thickening of the colon and the ileum (arrows) with enlarged mesenteric lymph nodes

An abdominal CT and abdominal MRI showed a circumferential wall thickening of the colon and the ileum (Fig. 1) and mesenteric fat infiltration suggestive of Crohn’s disease. It also presented enlarged mesenteric lymph nodes and a sessile polypoid mass of the rectosigmoid junction.

**Fig. 2:**
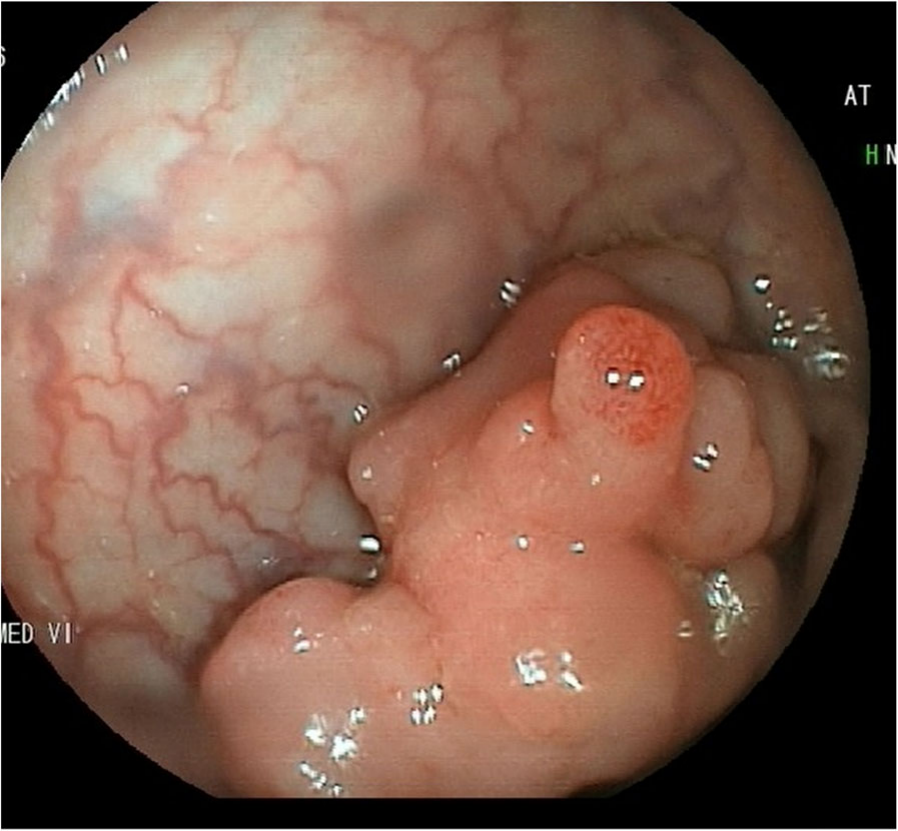
Colonoscopy showed a sessile polypoid mass at 17 cm from the anal verge

Ileo-colonoscopy revealed a 3 cm sessile polypoid mass at 17 cm from the anal verge (Fig. 2), many ulcerative and hemorrhagic lesions of the ileum and pseudo-polypoid appearance of ileocolonic mucosa.

The polypoid mass, the colonic and ileal mucosa were biopsied.

### Histological examination

The histological examination of the recto-sigmoid polyp showed a high-grade dysplasia with heavy mononuclear cell infiltrate suggestive of reactive lymphoid hyperplasia.

Histology from the colonic mucosa showed histologic features of Crohn’s disease with heavy mononuclear cell infiltrate suggestive of reactive lymphoid hyperplasia, while ileal biopsies showed a chronic ileitis without granulomas.

Discussion in the multidisciplinary meeting confirmed the presence of a polypoid high-grade dysplasia in a patient with Crohn’s disease. Due to the difficulty of a complete endoscopic resection and the multifocal nature of dysplasia in Crohn’s colitis a surgical removal of the colon was considered more appropriate. Consequently, the patient underwent an ileocoletectomy with resection of the upper rectum and ileorectostomy.

**Fig. 3:**
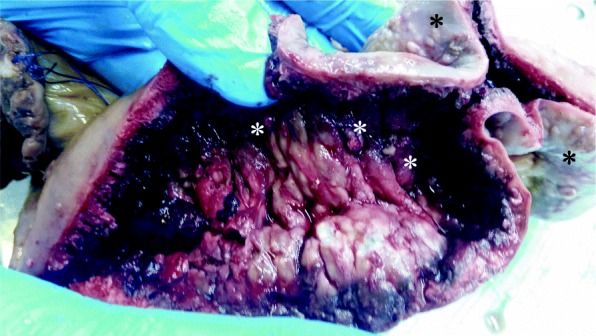
Surgical specimen: before formalin fixation showing numerous sessile polyps of varying sizes of the intestinal mucosa (white asterisk) with some ulcerations and whitish granulations in the ileum serosa (black asterisk)

Gross examination revealed a surgical specimen measuring 65 cm with a 3.5x2x2 cm polypoid mass at 5 cm from the surgical margin. Ileocolonic mucosa showed a multiple sessile polyps of different sizes (2–7 mm), ulcerations and granulations. The last characteristic was only seeing in the ileum serosa (Fig. 3). Multiple enlarged mesenteric lymph nodes were also found.

Pathology of the resected ileum revealed large, deep and discontinuous ulcerations without granuloma; there was also a diffuse lymphoid infiltrate that had reaches the serosa.

**Fig. 4:**
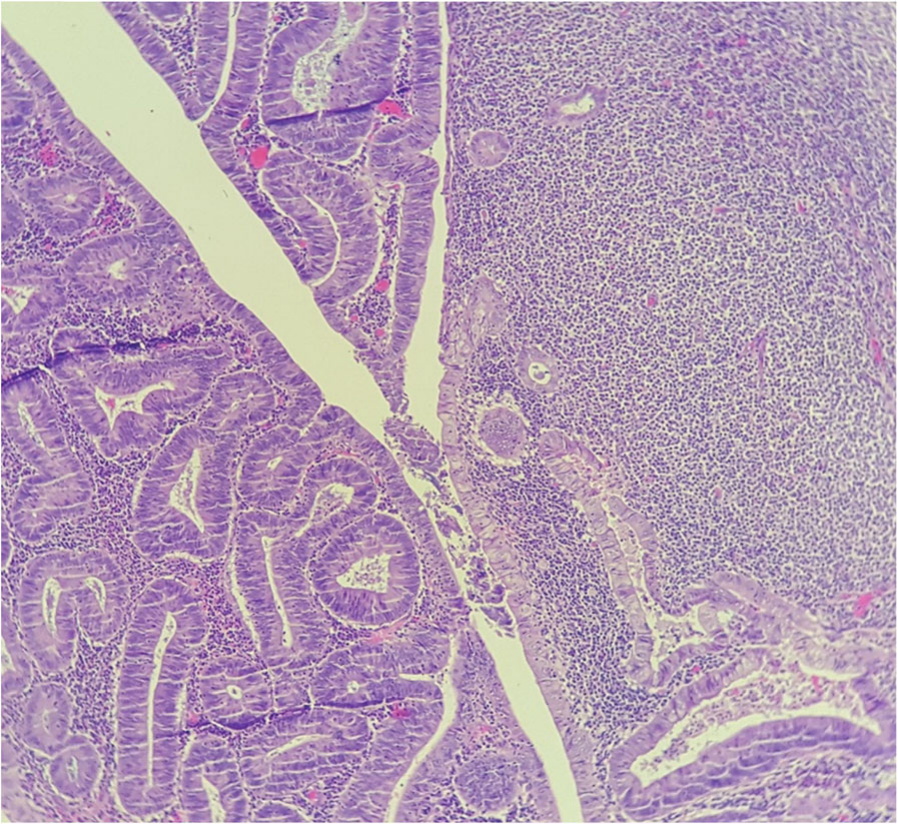
Adenoma with high grade dysplasia, and extensive small lymphocytic infiltrates at the base of the adenoma (HESx5)

The histological examination of the resected colon showed an adenoma with high grade dysplasia. Extensive small lymphocytic infiltrates were noted at the base of the adenoma (Fig. 4). We also noted 2 areas of low grade flat dysplasia.

**Fig. 5:**
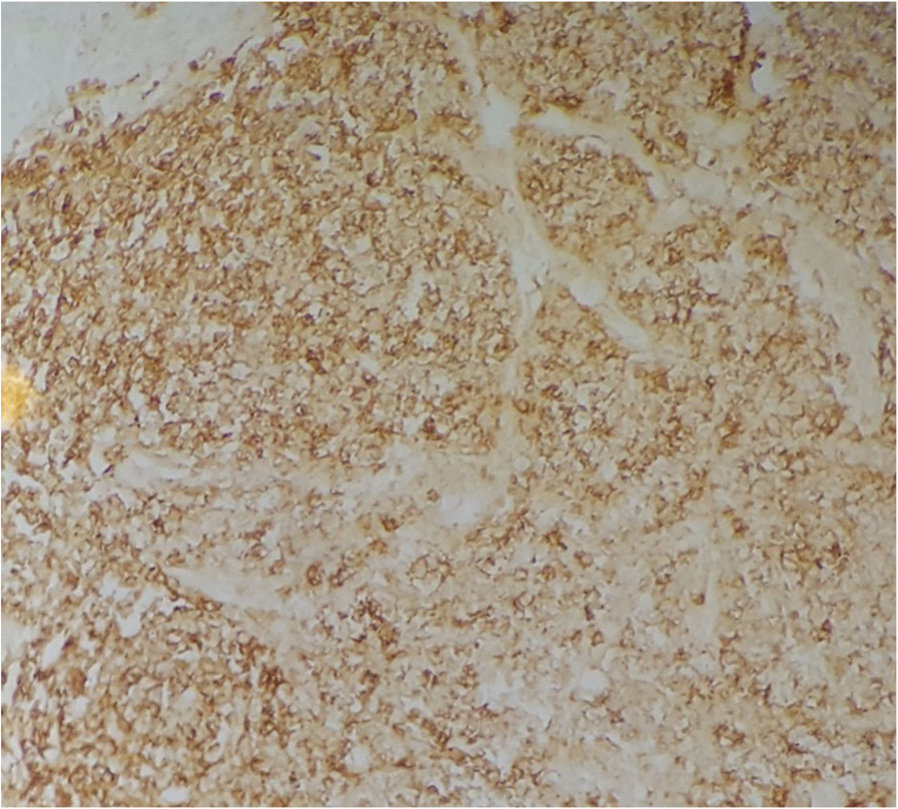
The small lymphocytes are strongly stained with CD 20

Immunohistochemistry of the lymphocytic infiltrates showed a strong and diffuse positivity for CD20 (Fig. 5), and BCL2, while CD3 highlighted some mature T-cells in the background. The CyclinD1, CD10, CD23 were negative. The diagnosis of colonic adenoma associated with MALT lymphoma in a background of Crohn’s disease was made.

Twenty-five lymph nodes were also invaded by the MALT lymphoma. The patient successfully completed 8 cycles of Rituximab+ chlorambucil chemotherapy.

Nowadays the patient is asymptomatic without evidence of lymphoproliferative recurrence 10 months after surgery.

## Discussion and conclusion

It is well known that patients with colonic Crohn’s disease have a high risk of developing colorectal cancer. This risk increases exponentially with the duration and extension of the disease [4]. The immediate precursor of CRC in IBD is dysplasia. In addition to their microscopic classification (low grade and high grade dysplasia), dysplastic lesions in IBD are also classified endoscopically as flat or raised also referred to by the acronym as DALM (dysplasia associated lesion or mass), which are further classified as resectable (‘adenomalike’) vs. non-resectable (‘non-adenomalike’) [6]. Endoscopic surveillance at regular intervals is the reference method for detecting theses lesions and carcinoma at an early stage [3], by using high-resolution technique combined with indigo carmine (or methylene blue) staining.

Patients with Crohn’s disease are also at increased risk of developing lymphoma. This association has been reported in many case reports. In 60% of these cases lymphoma in Crohn’s disease was localized in the small or large bowel and in 40% it was extra intestinal [7].

Although most of studies suggested that the use of immunosuppressive drugs, like infliximab, azathioprine, and 6-mercaptopurine, may increase this risk, many other studies reported patients who developed lymphoma without any immunosuppressive therapy other than corticosteroids [8–10]. This finding suggests that the increased risk could be only associated with the severity of the disease.

The occurrence of a lymphoma in Crohn’s disease patients can be explained by deregulated interactions between the immune system and the luminal bacteria which are implicated in the pathogenesis of Crohn’s disease [1]. It can also be related to chronic inflammatory per se, since it may lead to an in situ genesis of lymphoma in other contexts [11] . Since the discovery of new types of Th1 cells such as Treg, Th3, and Th17, as well as the application of antibodies to inflammatory factors TNF-α, IL-12, IL-22, and other new cytokines, it is clear that Crohn’s disease is a complex disease, with different mechanisms in different periods of disease progression, which is why the use of IL-12 or TNF-α antibody treatment in many patients with Crohn’s disease is not so effective [12].

Our case presents a possible insight into the interrelationship of Crohn’s disease and the immune system as seen with the coexistence of precursor lesion and lymphoma.

Several subtypes of lymphoma have been described in patients with Crohn’s disease: Hodgkin’s lymphoma, follicular lymphoma, non-Hodgkin’s (low grade lymphoma) [13], anaplastic large cell lymphoma [14], Hepatosplenic T-cell lymphoma [15], EBV-associated plasmablastic lymphoma [16], with only two cases of MALT lymphoma [13, 17].

MALT lymphoma is an extranodal lymphoma characterized by heterogenous small B-cells proliferation [18]. Stomach is the commonest site, while Colonic MALT lymphomas are exceedingly rare. In the current case, MALT lymphoma had a unique and rare presentation as multiple lymphomatous polyposis. Such polypoidal lesions can’t be differentiated from adenomatous or hamartomatous polyposis by colonoscopy alone. The histological examination with immunohistochemical study is mandatory.

We herein report an exceptional case of dual intestinal lymphoma and dysplasia associated with Crohn’s disease. Synchronous adenocarcinoma and lymphoma in patient with Crohn’s disease has rarely been documented [5]. However, we didn’t find any paper about the coexistence of precursors lesions and lymphoma in a patient with Crohn’s disease.

This case may be the first in the literature describing this rare association between a precursor lesions and a primary MALT intestinal lymphoma in the same surgical specimen.

Clinicopathological presence in multidisciplinary meetings to discuss such difficult cases is mandatory to reach the appropriate management.

## Data Availability

All the original data supporting our research are described in the Case presentation section and in the figures’ legends.

## Abbreviations

CT: Computed tomography
HES: Hematoxylin eosin safran
MALT: Mucosa-associated lymphoid tissue
MRI: Magnetic resonance imaging
